# Central Dogma Cycle and Network: A Model for Cell Memory

**DOI:** 10.1002/bies.70143

**Published:** 2026-05-12

**Authors:** Martin R. Schiller

**Affiliations:** ^1^ Nevada Institute of Personalized Medicine and School of Life Sciences 4505 S. Maryland Pkwy University of Nevada Las Vegas Nevada USA; ^2^ Heligenics Inc. 10530 Discovery Dr. Las Vegas Nevada USA

**Keywords:** Central Dogma, cycle, digital logic, memory, network

## Abstract

This paper proposes an extension of the traditional Central Dogma of molecular biology to a more dynamic model termed the Central Dogma cycle (CDC) and a broader network called the Central Dogma cyclic network (CDCN). While the Central Dogma is necessary for genetic information flow, it is insufficient to fully explain cellular memory, decision‐making, and information management. The CDC incorporates additional well‐established steps such as protein folding and networking, highlighting the cyclical nature of biological information flow. I propose that this cyclic architecture functions as a key mechanism for cellular memory, drawing analogies to memory functions in computers, such as input, read, write, execute, and erase. Within the CDCN, interconnected metabolic and signaling pathways act as logic‐enabled processors that bridge DNA mutations to phenotypes. This model reframes heredity beyond nucleic acid sequences and evolution as the optimization of memory‐bearing networks. It is extensible to broader biological systems such as physiological feedback loops. Understanding cellular memory through this cyclic network model offers a unified perspective on heredity, adaptation to the environment, cell processes, and the disruptions of information flow in disease pathology.

## Graphical abstract

This paper revisits the Central Dogma, expanding it to the Central Dogma cycle (CDC) and Central Dogma cyclic network (CDCN). These models frame cellular memory as a system of logic‐based processes with an architecture of interconnected cycles that more accurately describe cellular information flow in cells.

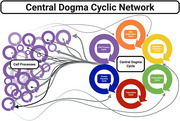

## 1 Introduction

**FIGURE 1:**
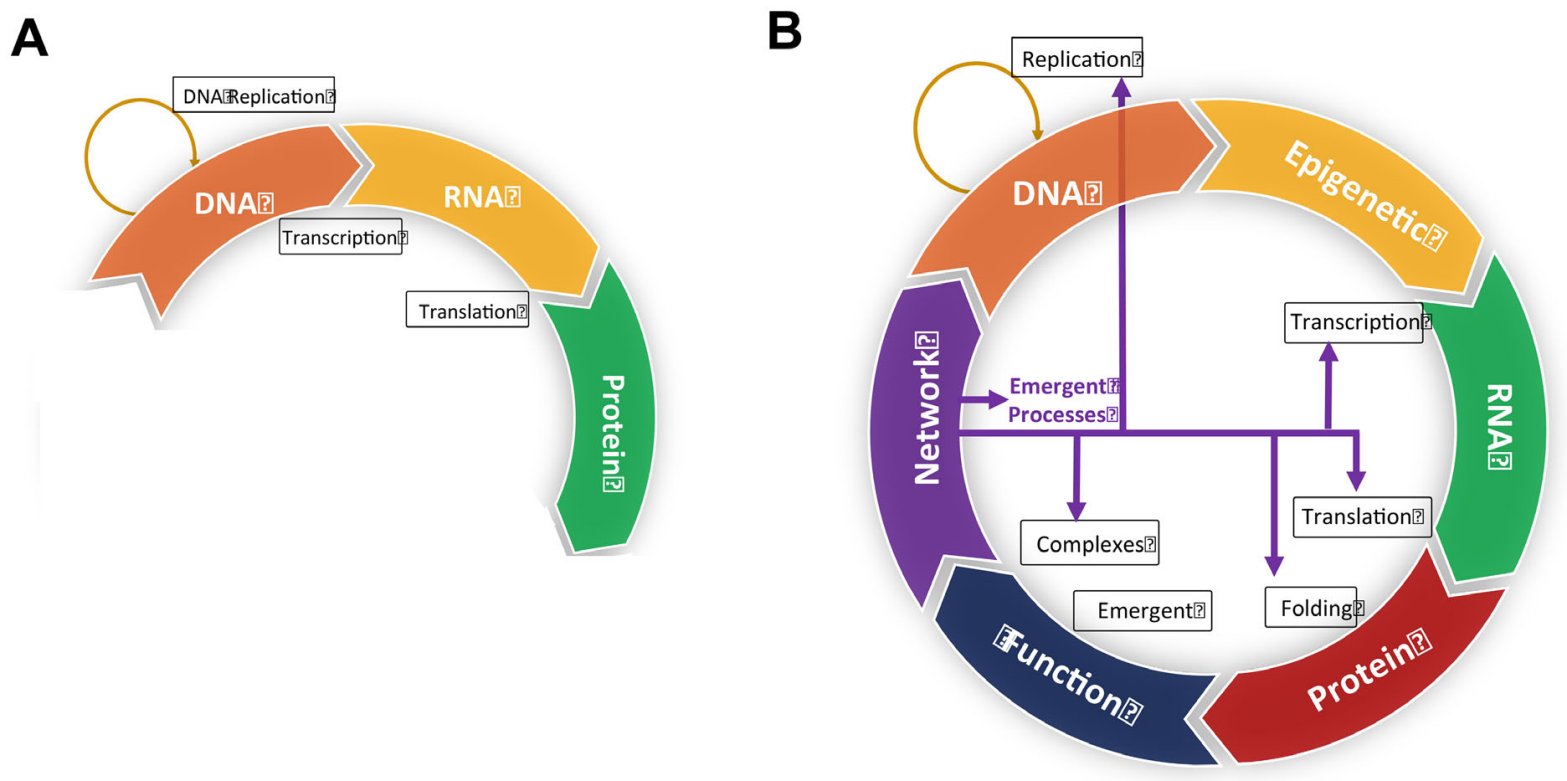
The CDC. (A) Established Central Dogma pathway for genetic information flow in cells. DNA is replicated or transcribed into RNA, which is subsequently translated into proteins. (B) The proposed CDC for information flow in cells. Expanding upon the Central Dogma in panel A, an epigenetic step (yellow box) is inserted between the DNA and RNA steps. This reflects how epigenetic enzymes, DNA modifications, and the modification of histones and nucleosomes allow chromatin to gate access to the DNA, thereby regulating transcription of RNA. After transcription, the translation of mRNA produces protein chains. Following the completion of the Central Dogma steps, many proteins undergo folding, acquiring specific biochemical activities and physical properties. The “function” (blue box) is an emergent property of the protein sequence, chemistry, and fold where the resulting activity is greater than the sum of its constituent parts. While the exact mechanism of emergence is not yet fully understood, it is a property derived from the primary, secondary, and tertiary structures of the protein. These functional proteins then form complexes with other proteins, macromolecules, and small molecules to combine into a network (purple box). This network drives essential cell processes necessary for metabolism, homeostasis, growth, and replication. Just as the molecular function emerges from the protein sequence and protein chain folding, the broader cell processes, including DNA replication, transcription, translation, and folding are emergent properties of the CDC network forming the cell processes that drive the steps in the cycle (purple arrows). In addition to the DNA, the entire CDC architecture is inherited by progeny cells and is part of the memory necessary for these core cell processes. The figures were created with PowerPoint.

The Central Dogma of molecular biology describes the flow of genetic information, whereby DNA replicates based on its sequence complementarity, is transcribed into an RNA copy of the DNA template, and the ribosome translates mRNA into a protein chain (Figure 1A). Originally proposed by Francis Crick in 1958, the “sequence hypothesis” indicates that the linear base sequence of nucleic acids codes for the amino acid sequence of a protein. He also postulated that such transfers from protein to protein, protein to RNA, or protein to DNA do not exist. Expanding upon these postulates, James Watson proposed the “Central Dogma” indicating a unidirectional information flow from DNA to mRNA to proteins, and once transferred to a protein, cannot be transferred back to nucleic acids [1, 2]. The Central Dogma has since been revisited several times [1, 2, 3, 4, 5, 6, 7, 8].

DNA can be considered a fundamental form of cellular memory that is transmitted to progeny cells. I redefine “cell memory” as: the encoding, storage, and retrieval of information in a living cell, which simplifies and unifies previous definitions for cell memory. However, DNA transfers only the nucleic acid and its sequence to daughter cells and is therefore insufficient to convey the entirety of cell memory. For example, aspects of cellular physiology encompassing many processes persist for months in mammalian enucleated red blood cells, despite their lack of DNA, nuclei, and mitochondria. This observation suggests the existence of previously unrecognized modes of cell memory inheritance beyond DNA.

In this paper, I explore the concept of cell memory, and examine potential mechanisms supported by findings in the literature. While the Central Dogma is a foundation for understanding information storage and transfer in cells, it is insufficient to explain how memories are preserved, transmitted to new cells, or even maintained in the same cell. Furthermore, the Central Dogma does not account for more recent discoveries such as epigenetic regulation of transcription, or the ways in which protein complexes drive cell processes that, in part, drive the transfer of information. To address these limitations, I propose extension of the Central Dogma to a more dynamic framework: the Central Dogma cycle (CDC), embedded within a broader network that I call the Central Dogma cyclic network (CDCN).

While metabolic and signaling pathways are a cornerstone of cell biology, the CDC and CDCN models move beyond mapping the flux of sequence information, defining a more complete architecture of cellular information management, similar to that for a computer. In this framework, molecular interactions are viewed as digital logic gates (e.g. AND or NOR) and organized into a collection of interconnected cycles. This molecular architecture of the cell manages environmental inputs, its state, and memory of events through orderly information processing.

## 2 Results

### 2.1 The CDC

Recognizing that the Central Dogma is not merely a linear pathway but a more complex set of interconnected cycles, I introduce the concept of the CDC as the hub architecture, a central engine for information flow in cells (Figure 1B). This cyclic architecture functions within the constraints of an open system. The ‘input’ function of the CDC (see Table 1) is subject to the thermodynamics of entropy, mass, and energy exchanges with the environment triggering the internal self‐organization of the network.

**TABLE 1:** Memory functions across human nervous system, computers, and cells.

Privileges	Nervous system	Computers	Cells
Input	Sensory input	Device input (keyboard, mouse, and sensors)	Protein factors, environmental conditions, and reproduction
Read (access and view)	Short‐term memory (hippocampus)	Read‐only memory (ROM), cache, RAM, and hard drive	DNA, chromatin, mRNA, Post‐translational modifications (PTMs), and epigenetic marks
Write (store and modify)	Long‐term memory, neuroplasticity, and synaptic changes	External memory (hard drive), cache, RAM, and hard drive	DNA, mRNA, proteins, PTMs, and epigenetic marks
Execute (move, duplicate, move, and copy)	Memory consolidation, synaptic plasticity, and gene expression	Processor, cache, and RAM	Signaling pathways/trafficking, replication, transcription, and translation
Erase (delete)	Short term memory loss and forgetting	Deletion from hard drive and RAM clearance	Degradation, apoptosis, PTMs, and epigenetic modifications

The Central Dogma can be conceptually extended into the CDC by incorporating additional well‐established steps. Since the discovery of the Central Dogma the significance of epigenetic regulation has become much better understood and is now included in the CDC as a fundamental step. In this stage, epigenetic enzymes modify DNA, histones, and nucleosomes, allowing chromatin to gate access to the DNA, and thereby regulate the “read” function of RNA transcription.

Following translation, and production of a protein chain, the next essential step for many proteins is protein folding. The pivotal experiments of Christian Anfinsen's and his colleagues demonstrated that a protein primary sequence carries the information necessary and sufficient for folding of a purified protein into a three‐dimensional (3D) structure [9]. While pure protein can fold in vitro, molecular chaperones assist folding inside the complex environment of cells.

Predicting protein folds has persisted as longstanding scientific challenge. While homology modeling was adopted as the first widely adopted method for predicting protein folds, it was limited to proteins having high sequence similarity that also had a previously known fold. The recent advent of machine learning approaches such as AlphaFold 2 has significantly enhanced predictive accuracy across proteomes [10, 11, 12]. However, despite these advancements and the knowledge that folding is driven by energetics, a precise deterministic code governing protein folding remains elusive. Notably, protein folding represents a form of inherited memory, as its sequence determines the fold, and the sequence is a transformation of the gene's DNA sequence.

In the next step of the CDC, folded proteins encode a wide array of specific biological functions including enzymes, binding proteins, and structural proteins. The fold typically determines the molecular function output. While some proteins do not adopt stable folds, instead remaining intrinsically disordered proteins (IDPs), resolving their functions is more challenging. Current machine learning tools such as PhiGnet, ProteInfer, and DeepGO and David Baker's work using machine learning for the *de novo* design of new protein folds are significant advances. However, a universal framework to predict the function of a protein from its sequence alone is in its infancy [13, 14, 15]. Notably, similar to the protein fold, the molecular function is a form of cellular memory that is also passed to progeny cells.

Predicting the emergence of protein function from sequence might benefit from a digital logic framework [16, 17]. Recent studies of intragenic epistasis (the dependent interactions of amino acids) demonstrate that all variants interact with discrete logical relationships modeled with digital gates. These findings suggest that digital gates may coalesce into circuits or networks of interactions functioning as computational circuits, akin to electrical circuits, where logic gates combine to produce and/or regulate functional output [16, 17, 18]. By extending this framework, the CDC models cell processes better than traditional metabolic and signaling charts to define a more precise architecture for cell information management (Figure 1B). This frames cellular memory as a product of the self‐referential, cyclic flow of information.

In the final step of the CDC, proteins with specific functions form a network with other macromolecules and metabolites, producing emergent cell processes that govern cell behavior. It is well established that protein networks possess numerous emergent properties, many of which are catalogs in the Gene Ontology database [19]. In my model, these emergent properties include the foundational processes that underlie steps in the CDC: DNA replication, epigenetic regulation, transcription, translation, protein folding, and protein networking. Each of these processes connects to a specific CDC step, closing a directed cycle. Crucially, complex biological processes arise from the coordinated activity and emergent properties of a network, rather than individual pathways or molecules. This creates a self‐referential and autopoietic feedback loop, in which the network produces the very machinery (such as DNA polymerase complexes or transcription factors), required to sustain its own cycle. Because living cells are thermodynamically “open systems,” this cycle must be understood as a dynamic process in constant flux with its surroundings.

By framing genetic information flow as a cycle rather than a unidirectional pathway, the CDC captures the intricate interdependencies that define living cells. This perspective shifts the traditional “matter‐flow” view, like that of signaling and metabolic charts to more precise information management architecture. The cyclic nature of the CDC provides a foundation for cell memory, integrating molecular biology and omics to construct a more comprehensive view of heredity. A mechanism is suggested  for the cell to retain, store, and retrieve knowledge about prior environmental inputs.

### 2.2 Comparing Memory Across Humans, Computers, and Cells

I digress to discuss the fundamentals of memory. DNA replication followed by mitosis or mieosis are the accepted primary mechanisms by which memory ‐ is transferred to progeny cells. This mechanism of inheritance is a form of memory, transmitting genetic information from one cell to another, and from parent organisms to progeny. Heredity is not traditionally called “memory”, which is more commonly associated with a brain or a computer. “Memory” is the capacity of a biological or artificial system to retain, store, and retrieve historical information.

Exploring the analogies of memory provides new insights into inheritance and information flow in cells. Cells inherit and transmit genetic information to many different functions, pathways, and processes not captured in the Central Dogma or the aforementioned CDC. For example, the MAPK pathway has short‐term memory, adapting its response based upon prior input [20].

There are five general memory functions conserved among the nervous system, computers, and cells: input, read, write, execute, and erase, although there are semantic differences (Table 1). By comparing memory across biological, computational, and neurological systems, we gain a new perspective about inheritance, information, and processing, which can inform on the broader concept of the memory usage in cells.

The input function receives information from an external source. Cells take inputs from external primary messengers or environmental conditions. The read function, also called access or view, accesses stored information without modifying it. In cells, genetic information is read from DNA and written by transcription and replication. Other forms of cellular memory have specialized reading, moving, executing, and writing functions, collectively called cell processes.

The write function, also somewhat synonymous with store or modify, reads existing memories as an input, and writes the information to a new location (store) or overwrites information at an existing location (modify). In cells, inputs are written in many different biochemical ways. The cell has multiple mechanisms for short‐term storage, such as chemical concentration gradients, capacitance, protein localization, post‐translational modifications of proteins, covalent and structural modifications of chromatin (epigenetic), and gene expression. Longer‐term storage is in the form of DNA.

The execute function reads stored information, transforms it, and writes the output to a location. Cells are more heterogeneous in how memory is processed. Cells execute many processes such as DNA replication, transcription, translation, or many others. The erase function, also called delete, removes information stored at a specific location by degradation. Long‐term memory in the form of DNA is generally not erased, similar to the nervous system.

### 2.3 Do Cycles Create Memory in Biological Systems?

**FIGURE 2:**
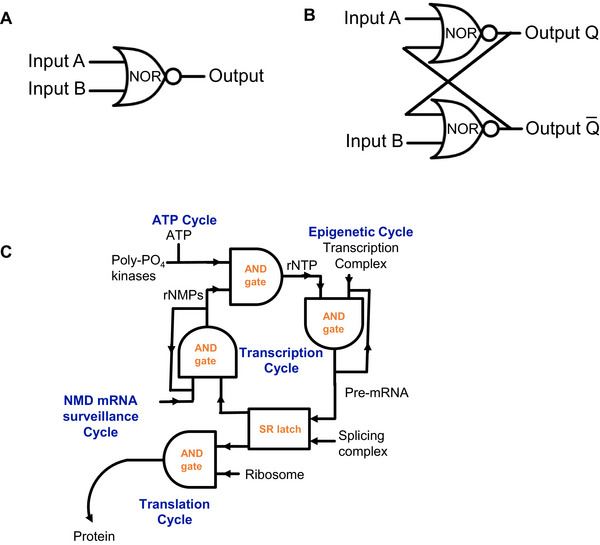
Memory encoding in computers and applied to the CDC. (A) A NOR gate only produces an output when both inputs are off. (B) One version of a bit (SR latch) is built from a circuit combining two NOR gates, each with two inputs and two outputs (A and B). One output of each NOR gate is one of the two inputs of the other NOR gate with Inputs A and B being the additional inputs for each gate. Output Q and its inverse, Q̄ are the outputs of each gate. 0 is considered no input and 1 is considered an input. The output behaviors of each input are dependent on the history of the other input. (C) An example expressing how the transcription cycle can be encoded by digital logic gates to create memory and make decisions. Cycles are labeled with blue fonts and electrical symbols with orange fonts. Four steps of the transcription cycle (transcription, pre‐mRNA splicing, RNA degradation, and RNA base regeneration), one step inputting to the translation cycle in the CDC, and the nonsensemediated decay (NMD) and ATP cycles from the broader CDCN are shown. The figures were generated with PowerPoint.

I question “How does a cell preserve memories that are not encoded in a nucleic acid sequence?” This question is addressed by considering an analogy to computer memory. When a cyclic circuit has an input that changes the output in response to a second input, this is the simplest expression of memory. In computers, the fundamental unit of memory is a bit, which can be represented by a “SR latch”, a flip‐flop two‐component circuit with two NOR gates and two switch inputs (Figure 2A,B) [21]. Similarly, a “D latch” is a oneinput form of memory, adding a simple circuit input to the SR‐latch. Computer memory is built from clusters, usually of 8 bits, combining them to form a “byte”, which can be sequentially combined to produce larger memory blocks. I hypothesize that organisms may have evolved a cyclical architectural framework within cellular systems that supports both the generation and storage of memory.

### 2.4 Metabolic Cycles

In the following sections, I review evidence consistent with thecycle hypothesis. Iacknowledge that cellular cycles likely serve multiple functions, and that additional, yet unidentified mechanisms of cellular memory may also contribute. Upon reviewing the architecture of molecular and biochemical networks, it is striking how many cellular events, including metabolic, signaling, synthesis versus degradation, or feedback loops, are embedded in cycles. While some processes appear relatively linear or unidirectional, the prevalence of cyclic organization suggests it is a foundational principle for biological information management.

In the 1930s, Albert Szent‐Györgyi and others identified several key metabolic reactions that were eventually recognized to be part of the Tricarboxylic Acid cycle for oxidative metabolism, also called the Krebs cycle; this is a cornerstone of the cyclic framework. The discovery that acetyl‐CoA condenses with oxaloacetate to citrate effectively closed these metabolic reactions into a cycle [22, 23]. Driven by input factors such as acetyl‐coenzyme A (CoA), nicotinamide adenine dinucleotide (NAD^+^), guanosine diphosphate (GDP), flavin adenine dinucleotide (FAD), and oxygen, this cycle not only processes energy, but maintains a cell‐based metabolic memory.

While the genes for the enzymes in these pathways are inherited via DNA, it is not yet clear how the molecules themselves or broader architectural cycles they constitute are inherited. Supporting this hypothesis is the existence of a “cell‐based metabolic memory” that persists in mammalian enucleated red blood cells throughout their 120‐day lifespan [24]. This suggests that cell‐based memory, extends beyond metabolism to other cell and molecular processes, and is passed from cell to cell and organism to organism, and is responsive to the environment. It is important to distinguish this from the clinical term “metabolic memory”, which refers to the persistent physiological state observed in Type 2 Diabetes patients after blood sugar levels are normalized [25, 26].

While the Krebs cycle is one of the earliest discovered and most well‐known biological cycles, cyclic organization is a pervasive feature in many cellular and physiological processes, as evidenced by over 88,000 papers identified by searching PubMed with the term “cycle” [27].

A cell can be modeled as a vast set of interconnected cycles (the CDCN) in which each cycle receives inputs from and produces outputs to other cycles in the cell. Many such cycles are already established in the literature. The urea cycle catalyzes nitrogen waste disposal, while fatty acid synthesis and beta‐oxidation form the cycles that control lipid metabolism [28, 29, 30]. Other cycles include the glyoxylate cycle, a variation of the Krebs cycle that converts fats into sugars, the Calvin cycle, essential for carbon fixation in plants and autotrophs, and the centrosome cycle regulating its duplication. This cyclic organization even extends to the environmental level, through the atmospheric carbon cycle converting inorganic carbon gases into organic molecules [31, 32, 33, 34].

Furthermore, energy‐carrying molecules like NAD^+^ and ATP are continuously regenerated through REDOX and the ATP cycles, respectively, forming the energetic drivers of many interconnected metabolic reactions [35, 36]. Because ATP and NAD^+^ drive numerous biochemical pathways, many metabolic processes involving these molecules inherently function as cycles. Typically, these metabolic cycles integrate with catabolic pathways that break down molecules into their constituents and anabolic pathways that utilize energy to synthesize new molecules.

### 2.5 Other Cell‐Based Cycles

Beyond metabolism, other biological cycles play critical roles in cellular and systemic function. The cell cycle regulates cell growth and division, while circadian rhythms govern biological clocks in nearly all living organisms [37, 38]. Other cycles are the secretory pathway: a series of organelle cycles involved in protein and membrane trafficking and secretion, and neurotransmitter recycling which ensures efficient communication between neurons by reusing neurotransmitters in synaptic terminals via a synaptic vesicle cycle [39, 40, 41]. Cycles are often identified by other names such as negative and positive feedback loops, such as those found in endocrine feedback regulation [42, 43]. Any cellular entity that undergoes a continuous process of synthesis and degradation operates within a homeostatic cycle, ensuring cellular balance and resource management; phosphoinositide signaling is a prime example of this maintenance [44].

This perspective highlights the importance of cyclic processes in maintaining biological homeostasis and adaptability. Given the sheer number of interdependent cycles, which likely number in the 10000s, a cell can be viewed as a vast CDCN. In this network, each cycle receives inputs and produces outputs for other cycles, creating a web of interactions. Importantly, the CDCN does not merely map the flow of molecules, as seen in traditional metabolic and signaling charts, but defines a logical architecture of information management.

A question arises: why has the cell adopted so many cycles as an organizing element? Some cycles help conserve metabolites, or regulate flow/energy through feedback [45]. However, a primary function of this architecture may be to serve as a mechanism for cellular memory, akin to how states are stored in computer memory. Each biological cycle has multiple inputs and outputs, making them functionally similar to a sequential circuit such as a SR latch or series of latches used in computer memory.

Significant evidence supports the encoding of a broader cell memory outside of the inherited DNA:

1. *DNA insufficiency*: Many critical cell processes continue in mammalian enucleated red blood cells for months, retaining the memory to perform complex physiology despite the total absence of nuclei, mitochondria, and DNA.
2. *Conservation*: Most cell processes and their underlying cycles are highly conserved across distantly related organisms, suggesting they are fundamental evolutionary solutions.
3. *Protein folding*: Proteins can fold *in vitro* based on their sequence alone, representing a form of memory that is independent of nucleic acids.
4. *Functional memory*: A protein fold holds a specific memory of molecular function that persists as long as the fold is intact.
5. *Inheritance of state*: During cell division, proteins, metabolites, organelles, and complete functioning cycles are passed to progeny, effectively inheriting the cells operational state.
6. *Architectural parity*: Cells possess architectural designs, specifically interconnected cycle networks that are computationally similar to RAM circuits in a computer.

By viewing the cell as a logic‐enabled processor, the CDCN framework of interconnected cycles provides a mechanistic basis for autopoiesis (self‐maintenance) and explains how biological information is preserved and managed beyond genomic sequences [46].

### 2.6 The CDC Network is a Framework for Cell Memory

**FIGURE 3:**
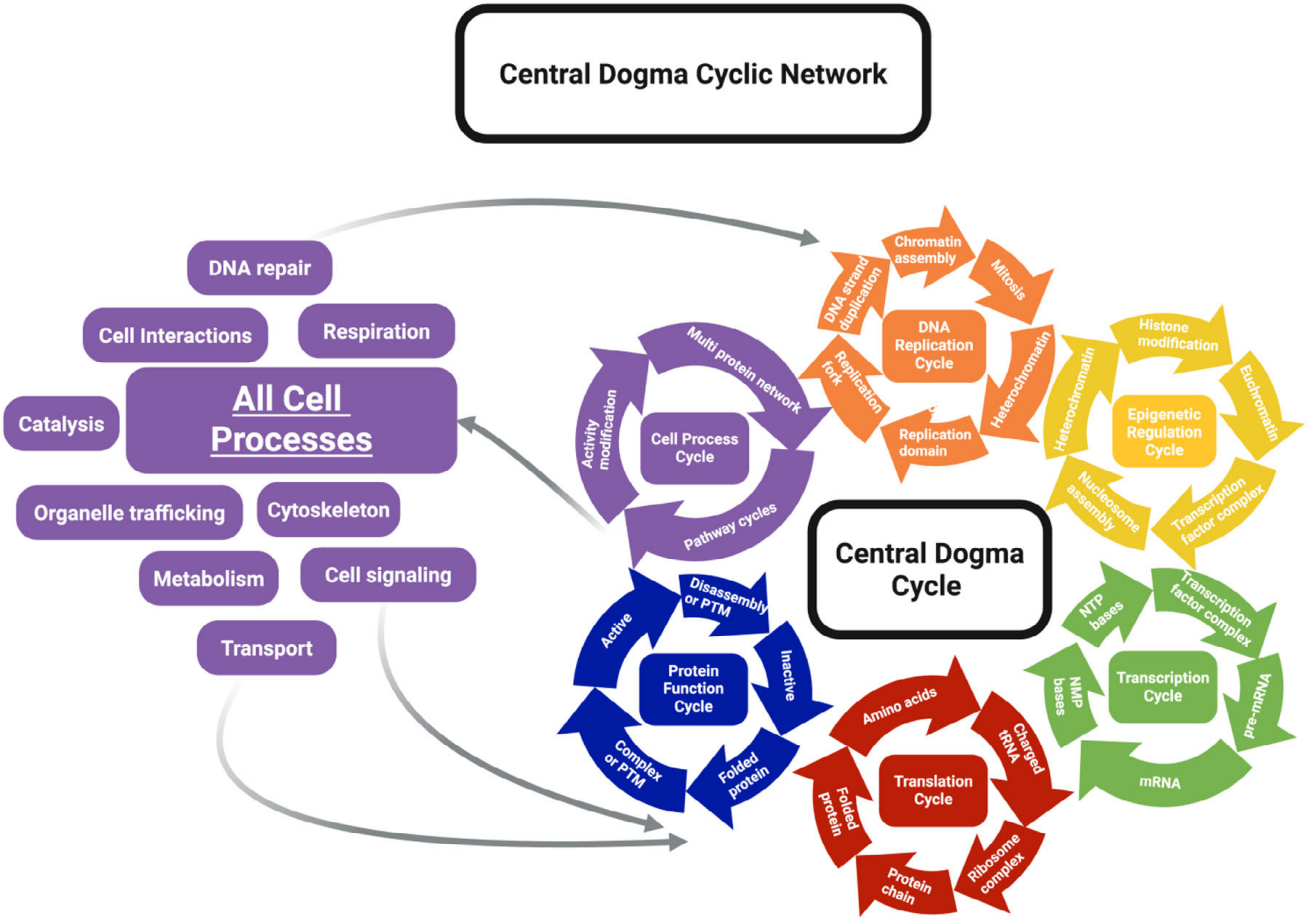
CDC and CDCN model. (A) This view of the CDC from Figure 1B breaks down each CDC step into a distinct cycle. The interconnected cycles are colored to match the corresponding steps in Figure 1B. In this representation of the CDC, the protein function cycle includes the functional state of the protein and the steps that regulate its function, such as addition or removal of posttranslational modifications, as well as degradation or disassembly of proteins and complexes. The network step from Figure 1B is relabeled here as the cell process cycle. The network of proteins, and other macromolecules produced as output of the CDC coalesces into a series of interconnected cycles shown as purple boxes on the left side of the diagram. Each box represents one or more cycles that combine, in which the numerous cell processes are emergent properties of this network. Some of these are shown as examples in the purple boxes. For example, the actin cytoskeleton involves a set of proteins that control the polymerization and depolymerization of actin filaments, a process that can be modeled as a cycle. The collection of these interconnected cycles is called the CDCN, which integrates the CDC as its core. Each cycle within this architecture can be considered memory of the associated cell process. Therefore, when a cell divides, these cycles are inherited, effectively inheriting the cell process itself. The figure was generated using BioRender.

The CDC was initially presented as a single informational cycle in Figure 1B. However, the CDC is a much more complex series of interconnected cycles organized within a higher order architecture called the CDCN (Figure 3). Core cell processes such as replication, epigenetic regulation, transcription, translation, protein folding, and protein function arise as emergent properties from these networked interactions. Once these processes emerge, they recursively drive other cycles within the CDC. For example, mRNA produced in the transcription cycle enters the translation cycle, coding for translation of specific proteins by the ribosome. This architecture functions similar to that of a computer's memory where individual cyclescan be viewed as interconnected bits that combine to form a larger functional unit of information, much like a byte unit of computer memory.

The individual cycles of the CDC correspond to specific memory functions. The DNA replication cycle is analogous to copying data for long‐term storage on a computer's hard drive. Access to the DNA for replication and transcription is gated by an epigenetic cycle regulating modifications of the DNA and nucleosome. This cycle acts as short‐term memory responsive to cell signaling or long‐term memory that can persist across generations. Transcription functions like the copy command; a gene is transcribed into pre‐mRNA and then processed into mature mRNAs, each of which has a variable half‐life serving as short‐term storage, akin to computer RAM. The resulting transcriptome represents a short‐term memory of which genes are actively expressed.

Due to their short half‐lives (hours or days), proteins function as short‐term memory like RAM, that can be quickly written, accessed and erased via degradation [47, 48]. Protein folding introduces an additional layer of short‐term memory. A correct fold is read through its emergent property, its specific molecular function, representing a transformation of memory from the linear sequence into a functional structure, analogous to storage in a hard drive. A folded protein has a precise memory for a specific function such as catalysis, light harvesting, binding small compounds, elasticity, and macromolecule interactions. While the DNA serves as a memory of the sequence, it does not inherently store the fold or the resulting function. Instead, a function is written from the protein sequence and its native fold, and these emergent functions are read by the cellular network to produce complex cell processes. As these processes are emergent properties of networks, they directly regulate progression through the CDC, including the recursive process of DNA replication.

Furthermore, these functions, pathways, and cycles are regulated by various post‐translational modifications. Because PTMs are dynamically added and regulated throughout the life of a protein, they represent a temporary, short‐term memory equivalent to a computer's cache. The cell processes emerging from the network are related to the execute function, serving as biological analogs to the processor in computers.

### 2.7 Do Some Steps in Cycles Mimic Latches?

Memory in biological systems can be modeled through fundamental digital logic constructs. A simple memory circuit can be formed with two NOR gates or two NAND gates, each with two inputs and one output (Figure 2A). In a NOR gate, when both inputs are off “0”, the output is on “1”; in all other cases where one or both inputs are on, the output remains off. Most circuits built from such gates are combinational, meaning their output depends solely on their current inputs.

In contrast, the SR latch is a sequential circuit, in which the output depends not only on the present inputs but also on the history of prior inputs, thereby creating a primary mechanism of memory. An SR latch is constructed from two cross‐coupled NOR gates, where the output of each gate becomes an input to the other (Figure 2B). In this configuration, external inputs A and B drive the circuit, while outputs Q and inverse Q̄ encode the specific memory state.

The SR latch operates in three principal states:

*Set*: Activating input A sets Q to 1, where it remains until a reset.

*Reset*: Activating input B resets Q to 0.

*Metastable (forbidden) state*: If both A and B are simultaneously active, the circuit enters an unstable forbidden state, with Q and Q̄ cycling continuously between 0 and 1 due to constant feedback. A video tutorial clarifies this unique behavior for biologists, who may be unfamiliar with the concept [49].

Applying these digital logic principles to biological systems, I modeled the Transcription cycle, a critical component within the CDC, using logic gates and memory elements (Figure 2C). The connection between transcription and mRNA decay was previously recognized to be cyclic [50]. By framing the connection between transcription and mRNA decay as a sequential logic circuit, we can better understand how the cell preserves informational states through its internal architectural design.

### 2.8 Initiation, Elongation, and Memory

A transcription factor complex input from the epigenetics cycle and ribonucleotide triphosphates (rNTPs) building blocks serve as dual inputs to a logical AND gate, producing pre‐mRNA as the output. Transcription is a progressive reaction, in which bases are sequentially added until the message is completed. This elongation phase requires molecular memory to sustain the continuous addition of bases; this is modeled by the output of the AND gate feeding back to the inputs (for simplicity, feedback to only one input is shown). When the first base is added, the output of 1 (active) is passed back to the inputs, ensuring the transcription cycle repeats iteratively. The feedback loop maintains the memory of the active state until a termination signal such as a hairpin loop causes the disassociation of RNA polymerase, resetting the gate to 0. Since both inputs must be present for initiation, the AND gate logic accurately reflects the biological requirement.

### 2.9 Splicing and Decision Making

Once pre‐mRNA is synthesized, it undergoes splicing to produce mature mRNA, a process requiring memory of the transcript to persist until synthesis is completed. This step is modeled by an SR latch, capturing two mutually exclusive outcomes: (1) Translation (combination with ribosomes via an AND gate, leading to protein synthesis in the protein cycle), or (2) Degradation (combination with RNases via an AND gate, leading to nonsense‐mediated mRNA decay in the NMD cycle). This mutual exclusivity is modeled by the paired inverse outputs of the SR latch, ensuring that the mRNA is either degraded or translated, never both simultaneously. The architecture mirrors the logic of the latch, which enforces a stable decision and prevents the forbidden state where simultaneous opposing actions of mRNA translation and degradation would compromise cellular integrity.

### 2.10 Recycling and Completion

When the mRNA synthesis is complete, it exits the Transcription cycle and enters the protein cycle. Then the mRNA combines with the ribosome, a step modeled as an AND gate, requiring both inputs to produce a protein chain. Alternatively, if the mRNA molecule is targeted for degradation, it enters the NMD cycle. Here, mRNAs are substrates for RNases in an AND gate, releasing ribonucleotide monophosphates (rNMPs) after digestion. Because degradation is a progressive process requiring the sequential removal of bases, it is modeled as an AND gate with feedback, mirroring the iterative logic of the transcription elongation step.

The rNMP substrates combine with polyphosphate kinase enzymes and ATP, replenishing rNTPs through an AND gate for the next round of transcription. The transcription and mRNA decay processes are mechanistically linked in cells [51].

Throughout these steps, memory is maintained by feedback mechanisms and logical constructs ensuring continuity until an explicit reset condition is met. Furthermore, external cycles interact with this system to regulate the flow of information, acting as signals that gate the cycle's progression.

Biological cycles may fundamentally operate using logic‐based architectures, where feedback loops, sequential progression, environmental inputs, and decision‐making nodes are embedded forms of cellular memory. The memory is distinct from, but complementary to the information encoded in linear macromolecular sequences. The logic gate construct applied here to the transcription cycle is likely similarly applicable to other cycles. This architecture offers a general way by which cells process and store memory outside of genomic coding.

For example, the Krebs cycle is also composed mostly of AND gates connected in a 10‐step cycle; both enzymes and substrates are required for each step. Crucially, it also features key decision points, to continue anabolic use or redirect its metabolites to synthesize amino acids and other molecules through connected cycles; this could be modeled by a latch. These decision points allow the cell to stably switch states between catabolic energy production, or divert intermediates for anabolic biosynthesis, thereby acting as a logic‐driven memory fullfiling the cell's metabolic needs.

### 2.11 The Central Dogma Cyclic Network

While the CDC serves as the information hub, it is just one of many interlinked cycles that arise from functional protein networks. Each of these cycles connects with others, including the CDC, forming dynamic and interconnected network architecture. In this framework, the output of one cycle often serves as the input for another, creating a robust web of cycle‐to‐cycle communication.

This network encodes a distributed form of biological memory. By collectively producing the protein complexes responsible for catalysis, regulation, and essential cellular processes, the CDCN allows the cells to be responsive to the environment. Consequently, each cellular behavior is an emergent property arising from the activity of one or more cycles, serving as a mechanism for memory of all cell processes that function alongside the inheritance of the gene sequences. As illustrated in Figures 2C and 3, these interconnected cycles likely represent evolutionary solutions to the requirements of autopoiesis, ensuring that cellular systems remember, reproduce, and regulate key processes reliably over generations.

The broad scope and significance of such cycles are clearly evident in signal transduction pathways. These pathways frequently use kinases as “writers” by phosphorylating target proteins. These modifications are subsequently “read” by adaptor proteins like 14‐3‐3, or those containing SH2 or PTB domains, strictly in a phosphorylation‐dependent manner. Completing the cycle, phosphatases act as “erasers” removing phosphate groups and restoring the protein to its basal state [52, 53]. Throughout this phosphorylation cycle, the functional output of the modified protein is modulated, thereby influencing downstream processes. Given the vast diversity of kinases and phosphatases, alongside the multitude of potential phosphorylation sites, it is likely that thousands of such signaling‐regulatory cycles operate concurrently within a cell, each interacting with and modulating others.

An analogous form of cyclical regulation governs the histone modification system and epigenetic memory. This system comprises enzymes that covalently modify histone tails (“writers”), proteins that recognize and bind these modifications (“readers”), and enzymes that remove them (“erasers”) [54, 55, 56, 57, 58, 59]. Emerging evidence suggests that cell signaling serves as a critical input into this epigenetic cycle, modulating histone marks and triggering downstream events such as nucleosome disassembly and the initiation of the transcription cycle. Consequently, environmental cues and signaling events that alter these marks contribute to short‐term epigenetic memory, distinctively shaping gene expression patterns over time.

The pathways and cycles delineated in this paper are presented in a simplified manner to illustrate key concepts. *In vivo*, cycles are substantially more complex, involving additional intermediary steps, extensive crosstalk with other cycles, and more sophisticated regulatory logic. While specific molecular interactions and sequential steps remain subject to refinement by subject matter experts, the central hypothesis emphasized herein is the functional significance of the cyclic architecture and its capacity to encode cellular memory.

## 3 Discussion

The model extends the Central Dogma to the CDC and CDCN, emphasizing the role of interconnected molecular cycles in establishing cellular memory. The DNA sequence alone is insufficient to establish the complex memories required for a cell to inherit function from its parent. Instead, cellular memory is maintained through emergent properties arising from proteins, functional pathways, molecular complexes, inter‐cycle connectivity, and their integration with small molecules.

Modeling the CDC and CDCN with cycle‐driven memory offers four distinct advantages when compared to the Central Dogma: (1) These models open novel avenues of inquiry regarding cellular information processing and inheritance. (2) They disambiguate and model cell reactions and processes with greater accuracy. (3) The framework is inherently extensible, accommodating all 16 possible binary logic gates to better represent regulation and homeostasis; and (4) The CDC and CDCN frameworks can accommodate and describe the full complexity of biological information processing.

This model also brings to light fundamental questions that have previously gone unaddressed. A prime example is the decision‐making logic a cell employs to determine whether to degrade or translate an mRNA molecule. If degradation and translation were to occur simultaneously, partially translated mRNAs would be destroyed, compromising protein synthesis. I hypothesize that this mutually exclusive decision is governed by a molecular regulatory circuit analogous to an SR latch (Figure 2B). This hypothetical bistable switch warrants direct experimental investigation. Notably, despite over 6 decades of research into the Central Dogma, the specific regulatory logic governing this critical bifurcation remains largely unexplored in the literature.

Existing models and diagrams of signaling pathways and cellular processes often fail to capture the necessary regulatory complexity, a deficit clearly seen in standard depictions of the transcription cycle (Figure 2C). Most pathway diagrams are limited to basic logic gates (OR, AND, and NOT); however, systems with two inputs and one output have 16 possible digital logic operations. A complete toolbox that embraces the full logic range is required to model progressive reactions, timing mechanisms, and memory formation; these are all critical aspects of biological behavior. Supporting this view, our recent work confirms that combinations of transcription factor mutations can produce all possible logical outputs, providing empirical evidence for the use of logical encoding in nature [16].

In addition to functional memory in mammalian red blood cells, a complementary line of evidence supporting the CDC and CDCN as a DNA‐independent memory system comes from bottom‐up research on artificial cells. DNA alone is never sufficient to produce a functional cell. Instead, researchers must introduce non‐genetic components including lipid or polymeric membranes that define cellular boundaries, cell‐free transcription–translation systems containing ribosomes, tRNAs, translation factors, metabolic cofactors, and ATP‐regeneration substrates [60, 61, 62, 63]. In some cases, structural components are required to stabilize morphology and mechanical signaling [64, 65].

These observations demonstrate that persistent cellular memory arises from the integrated biochemical network with modifying environmental inputs, rather than from DNA alone, enabling consistent behavior in artificial cells. This supports the hypothesis that the CDC and CDCN transmit a form of biochemical memory that, independent of DNA sequence, is thus far, required for artificial cells.

### 3.1 Potential Role of the CDCN on Evolutionary Biology

Traditional evolutionary theory centers on mutations and natural selection, yet studies of genetic polymorphisms alone often fail to explain complex phenotypic outcomes. The CDCN adds a critical dimension by introducing short‐term cellular memory, cycle inheritance, and logic‐based information encoding. This reframes evolution as the optimization of interlinked memory‐bearing networks that directly link DNA mutations to selectable traits. In this view, evolution operates through dynamic processors with emergent computational properties, similar to distributed interactions in AI large language models. The non‐genetic memory encoded by the CDC and CDCN likely originated before thelast universal common ancestor (LUCA). These cycles were likely subsequently inherited by LUCA's descendants as a stable, self‐perpetuating biochemical network architecture that has persisted in all progeny cells with adaptations from the environment. Validation of the CDCN would suggest that evolution shapes systems as adaptive information‐processing networks rather than simple replicators.

### 3.2 Cyclic Mechanisms of Memory May be Extensible to Other Systems

This cyclic model of memory is extensible, likely applying beyond the cell to other biological systems. It offers a broader framework for understanding how memory is created and managed across living systems.

Cycles are well‐recognized as a staple of physiology and cell communication. With approximately 300 different receptor types per cell, the potential for cyclic communication networks is vast [66]. For example, in response to stress, the hypothalamic‐pituitary‐adrenal (HPA) axis feedback cycle illustrates this: stress triggers a loop in which the hypothalamus secretes corticotropin‐releasing hormone (CRH), stimulating the pituitary gland to release adrenocorticotropic hormone (ACTH), subsequently stimulating the adrenal glands to secrete cortisol. Cortisol then provides negative feedback to the hypothalamus, completing the cycle. This establishes systemic memory, preparing the body for stress. Similarly, cyclic circuits likely govern memory storage and retrieval.

Beyond individual organisms, ecosystems depend on the carbon cycle for homeostasis between plants and animals. Plants capture sunlight energy, converting absorbed carbon dioxide into carbohydrates, releasing oxygen in the process. Animals consume these carbohydrates and oxygen to extract energy, returning carbon dioxide to the atmosphere through respiration. This cycle encodes ecological memory of how solar energy sustains energy balance in ecosystems.

Medically, disease can be viewed as disruptions in these memory cycles. Dysregulation of these information‐processing systems leads to homeostatic failure. Viewing disease pathology through this lens, suggests new therapeutic strategies focused on restoring normal information flow and cellular function.

### 3.3 Central Dogma Cyclic Network

## Conflicts of Interest

There is a potential conflict of interest as Martin R. Schiller is both a Professor at the University of Nevada, Las Vegas and an employee of Heligenics Inc., which produces and commercializes mutation effect on gene activity (MEGA)‐maps produced with the GigaAssay technology.
